# Experiences of symptom burden among young children born with esophageal atresia–tracheoesophageal fistula: a US focus group study

**DOI:** 10.1186/s13023-025-03939-2

**Published:** 2025-08-18

**Authors:** Michaela Dellenmark-Blom, John Bennett, Rosella Micalizzi, Lianne Cole, Kaylee Woods, Lauren Cardoni, Leah Frain, Abdimajid Mohamed, Jessica Yasuda, Peter Ngo, Anke Widenmann, Graham Slater, Benjamin Zendejas

**Affiliations:** 1 Department of Pediatrics, Institute of Clinical Sciences, University of Gothenburg, 416 85 Gothenburg, Sweden; 2 Department of Pediatric Surgery, Queen Silvia Children’s Hospital, Sahlgrenska University Hospital, 416 85 Gothenburg, Sweden; 3 Esophageal and Airway Treatment Center, Department of Pediatric General Surgery, Boston Children’s Hospital, Harvard Medical School, Boston, MA USA; 4 Division of Gastroenterology, Hepatology and Nutrition, Boston Children’s Hospital, Boston, MA USA; 5 EAT (Esophageal Atresia Global Support Groups), Sommerrainstr. 61, 70374 Stuttgart, Germany

**Keywords:** Esophageal atresia, Rare disease, Symptom burden, Patient-reported outcome, Tracheomalacia, Tracheobronchomalacia

## Abstract

**Background:**

Children born with esophageal atresia–tracheoesophageal fistula (EA–TEF) can suffer from aerodigestive morbidity that impairs their quality of life and can persist into adulthood. Ameliorating their symptom burden requires a thorough understanding of the symptom experiences that children have early in life. We aimed to explore parents’ experiences of their children’s aerodigestive symptom burden during the first years of life after being born with EA–TEF. This exploration also aimed to help determine whether a disease-specific measurement of symptom burden is needed.

**Method:**

Five standardized focus groups (FGs) with 22 parents of children with EA–TEF aged 6 months–7 years treated at a US tertiary pediatric surgical center were used to explore the children’s symptom experiences. The FGs were audio-recorded, transcribed, content analyzed into what symptoms were expressed, together with their stated frequency, severity and relation to child distress.

**Results:**

Twenty-two parents made 450 unique statements about their children’s aerodigestive symptom experiences. The respiratory symptoms (n = 170 statements, n = 21 parents) included the following unique symptom expressions; Breathing difficulties (n = 21), Breathing sounds (n = 6), Cough (n = 17), Mucus problems (n = 22), Prone to frequent or severe respiratory infections (n = 20) and Reduced physical capacity/strength (n = 8). The digestive symptoms (181 statements, n = 21 parents) encompassed symptom expressions of Acid reflux/heartburn (n = 7), Hiccups (n = 1), Nausea (n = 2), Reflux/food coming up (n = 10), Stomach problems (n = 4), Swallowing difficulties (n = 24) and Vomiting/throw-up (n = 6). The descriptions of respiratory and digestive symptom experiences included a variation of symptom frequency, severity and child distress. Furthermore, feeding difficulties (99 statements, n = 22 parents) included the children’s Food refusal (n = 8), Need for mealtime adjustment (n = 7), Selective/restrictive eating (n = 14) and Upset/stress with feeds (n = 10). Most parents (n = 20, 91%) described that their children had symptom experiences that spanned all three categories (respiratory and digestive symptoms, feeding difficulties).

**Conclusions:**

Young children born with EA–TEF experience a significant symptom burden that can be reflected as a summative composite of the dimensions respiratory and digestive symptom frequency, severity and distress, in addition to feeding difficulties. This supports the need for a disease-specific measurement of symptom burden that is guided by the content and wording obtained directly from the parents’ descriptions to help establish its content validity.

**Supplementary Information:**

The online version contains supplementary material available at 10.1186/s13023-025-03939-2.

## Background

Esophageal atresia (EA) is a congenital malformation of the esophagus and airway. EA refers to a discontinuity of the esophagus and presents in different anatomical subtypes. The anatomical subtypes of EA are commonly presented in relation to the presence and location of a tracheoesophageal fistula (TEF) using the Gross classification system. EA is rare and has an overall occurrence of 2.4 of 10.000 live births [1]. The prevalence of the anatomical subtypes varies across the spectrum: isolated EA (Gross A, 7–8%), EA with a proximal TEF (Gross B, 1–4%), EA with distal TEF (Gross C, 82–85%), EA with proximal and distal TEF (Gross D, 3–4%) and TEF only (Gross E; 3–4%). In around 55% of the cases, children with EA have associated anomalies, frequently pertaining to the cardio-vascular, gastro-intestinal, uro-genital and musculoskeletal system [2].

Most infants born with EA undergo a primary anastomosis to restore esophageal continuity within their first days of life, and if present, closure of a TEF. Around 10% of children with EA present with a gap between the two esophageal ends that is too long (long-gap EA, LGEA) [3, 4] to achieve a primary anastomosis, posing the need for alternative surgical strategies. LGEA is often managed by inserting a gastrostomy for enteral feeding, allowing for spontaneous growth of the esophageal segments, then performing a delayed primary anastomosis when the child is a few months old [5]. When the proximal and distal esophageal segments are too far apart for primary repair, the native esophagus can also be successfully preserved by utilizing “traction-induced growth” such as in the Foker technique [6, 7]. Esophageal replacement (ER) can be employed in cases of LGEA or failed EA repairs. The conduit of choice for ER varies among stomach, jejunum or colon and is highly dependent on the center where procedures are performed [3, 4, 8].

Today’s survival rates of the children with EA–TEF have reached almost 90% in high-income countries [9]. However, children with EA–TEF suffer from chronic aerodigestive morbidity [2], including dysphagia (55–70%) [10, 11], gastrointestinal reflux disease (GERD) (46–55%) and respiratory symptoms (52–69%) [11, 12]. In EA–TEF, a common source of respiratory morbidity is tracheobronchomalacia (TBM) contributing to cyanotic spells, chronic cough, recurrent lower respiratory tract infections, “wheezing”, impaired airway clearance, aspiration and bronchiectasis [13]. Both esophageal and respiratory morbidity can contribute to feeding difficulties in children with EA–TEF [2], which has been observed in 75% of the children up to 7 years of age [14]. In children with EA–TEF, the presence of aerodigestive symptoms substantially contributes to reduced levels of their health-related quality of life (HRQOL) and such morbidity can persist into their adulthood [15–18]. Therefore, prevention and amelioration of symptom burden in children with EA–TEF are important endpoints for patient-centered monitoring, care and treatment interventions, but requires a thorough understanding of the children’s symptom experiences early in life.

A patient’s symptom experiences fall within the definition of a patient-reported outcome, which can be evaluated using a valid and reliable patient-reported outcome measure (PROM). A PROM is a questionnaire that collects health outcomes directly from patients, or in pediatric patients, also from their parents. A PROM should reflect items reported as important to patients and parents in their own words. PROMs can facilitate the systematic collection of patients’ health to support health-care decisions, complementary to other evidence of clinical outcomes and/or biomarkers [19]. Drawbacks of previous studies investigating symptom experiences in children with EA–TEF is the use of non-instrumental methods, such as only asking parents a categorial question of the presence of symptoms in the past week or month [11, 14, 20, 21] or use of unvalidated author-generated tools [22–24]. A few studies have employed validated scales to estimate the children’s feeding problems or swallowing dysfunction in children with EA–TEF [25–30], but some of these studies applied instruments that were originally developed for adults or not specifically adjusted to EA–TEF [28–32]. Moreover, qualitative research is used to capture individuals’ descriptions of their experiences of a particular topic of investigation and can aid item generation of a PROM. In the field of EA–TEF, focus groups with patients with EA–TEF and their parents have been successfully used to understand their HRQOL experiences in more depth and yield item generation of disease-specific measures [33, 34]. Three qualitative studies have employed individual interviews with only a few parents of children with EA–TEF; three mothers [35], six parents [36] and eight mothers [37]. However, none of these studies have specifically focused on their children’s respiratory morbidities nor a comprehensive evaluation of their overall symptom burden.

Responding to the scarcity of research in this field, this study aimed to explore parents’ experiences of their children’s aerodigestive symptom burden during the first years of life after being born with EA–TEF, including parents’ descriptions of the symptom characteristics. Second, this exploration aimed to help determine whether a disease-specific measurement of symptom burden is needed in children with EA–TEF at an early child age and if so, aid such a development as a next step.

## Material and methods

### Study design

Descriptive qualitative study utilizing focus groups with a combined deductive-inductive approach.

### Ethical considerations

Ethical approval was obtained from the Institutional Review Board (IRB-P00048407, IRB-P00646718). Written informed consent was collected from the legal guardians prior to study participation.

### Framework

This study aligns with the US Food and Drug Administration Patient-Reported Outcome (PRO) standards, which define a PRO as any report of the patients’ health condition that comes directly from the patient, without interpretation of the patient’s response by a clinician or anyone else [19]. “Symptom experience” is considered a PRO concept, distinct from objective signs of disease [38, 39]. Hence, in this study, a symptom was regarded as the subjective evidence of disease or physical disturbance experienced by a patient, underlining that symptom experiences have a *negative* nature. A symptom experience consists of multiple dimensions, including the individual’s perception of the symptom frequency, intensity, severity, and distress [39, 40]. In turn, symptom distress refers specifically to the physical or mental suffering caused by a symptom. Individuals rarely experience isolated symptoms, but in combination or cluster with other symptoms. The term “symptom burden” refers to a collective multidimensional subjective counterpart of summary expressions of disease or treatment upon the patient [38, 39]. For infants and young children, a parent’s “observer report” of events or behaviors of their children are essential for capturing symptom experiences [19, 41].

### Setting and participants

Boston Children’s Hospital (BCH), a tertiary pediatric surgical centre specializing in multidisciplinary care of infants, children and young adults with complex esophageal and airway disorders, served as the study site. Eligible participants included parents of children born with EA of any of the anatomical subtypes Gross A, B, C, D or E who were fluent in written and oral English. At the time of the study, the child had to be between 0 and 7 years old, live at home and not be within the early recovery phase (first 30 days) of a major surgical intervention.

The goal was to recruit four to six parents to each focus group. To ensure a representative sample and increase group homogeneity among participants as recommended for successful focus groups [42], the focus groups were stratified by child age (0–2 years, 3–7 years) and the type of surgical repair: primary anastomosis, complex repair, including major revisional surgery or delayed primary anastomosis and ER. Stratification of the focus groups according to ER started at age 3–7 years because of the study center’s treatment strategy preference to avoid early ER in children with EA for the purpose of esophageal preservation. This meant that children living with ER were of an older age.

**Fig. 1:**
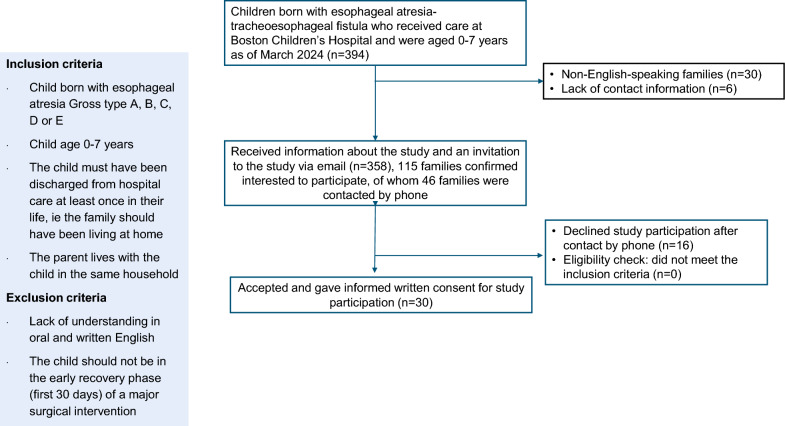
Flowchart of the recruitment procedure to five focus groups with parents of children born with esophageal atresia–tracheoesophageal fistula

Figure 1 presents a flow chart of the process used to recruit parents to the focus groups. Children born with EA–TEF (n = 394) were identified through a clinical database. All families received individualized email invitations sent through a center-specific distribution system which gave families the possibility to confirm interest for one parent in each family to participate. One hundred and fifteen parents responded to the email invitation. Forty-six purposively selected families matching the inclusion criteria were contacted by phone by a researcher to optimize representativity in the study sample. All families who confirmed interest to participate in the study were checked for eligibility according to the pre-defined inclusion and exclusion criteria. Thirty families accepted study participation and gave written informed consent for study participation. Participants received compensation via gift cards upon completion of the focus group ($100 for in-person groups, $50 for virtual groups).

### Data collection

A semi-structured focus group manual was developed to standardize the study procedure. To fully explore the parental perspectives of the child’s symptoms experiences in depth, the interview manual was developed based on previous content analysis of ten focus groups involving families of children with EA–TEF in Sweden [33] (n = 30) and input from a multidisciplinary research committee, with representation from an international EA support group. The interview manual covered three disease-specific domains for symptom experiences in EA–TEF: (a) Swallowing difficulties/Feeding/Dumping; (b) Gastro-esophageal reflux disease; (c) Respiratory disease, each with listed open-ended, probing sub-questions and follow-up questions (Additional file 2). The parents completed a socio-demographic questionnaire, and medical records were reviewed for neonatal and surgical history. Focus group discussions were facilitated by a trained moderator (MDB) with no prior clinical relationship to the participants, who ensured that all participants had an opportunity to contribute to the discussions. A field assistant (JB, LC, RM) documented non-verbal cues and group interactions.

### Data analysis

Focus group discussions were audio-recorded and transcribed verbatim. All symptom experiences were analyzed using the principles of manifest content analysis, as this focuses on explicit textual content of experiences described by participants themselves and on identification of categories sharing a commonality. All sub/categories were to be internally homogeneous and externally heterogeneous for content, meaning that no data should fall into more than one category/subcategory [43].

One researcher (MDB) read all transcripts of the focus groups, extracted all meaning units of aerodigestive symptom experiences, merged them with participant information into Microsoft Excel, condensed the meaning units and re-read the transcripts to check that the content had been covered in relation to the study aim. As a symptom experience is subjective evidence of disease or physical disturbance, parents’ statements that their children did not experience a symptom were not included in the content analysis. Using an inductive approach, this researcher continued to sort the condensed meaning units into codes, subcategories and categories, answering the question “what symptom was being expressed”. Each subcategory of a symptom experience was deductively analyzed in relation to four main dimensions (a) symptom frequency (b) when the symptom was expressed (situational context) (c) symptom severity (ie, parents’ expressions of the amount/degree of the symptoms, symptom intensity, symptoms leading to new care needs/consequences, symptom progression) and (d) symptom distress. When any of these symptom dimensions (a-d) were not identified in the parents’ statement, they were labelled as not reported. The procedure is exemplified in Additional file 1. The categorization process was critically and independently reviewed by a second (LC) and a third researcher (BZ) and discussed with the research team until consensus was achieved.

The focus groups design permitted study participants to stay with discussion topics important for them [42, 44]. By combining the quantification of a category, the magnitude of the symptom experiences can appear more clearly [43]. Therefore, descriptive statistics (n, %) were used to identify the participants who added to each given category of symptom expressed, and this was combined with the statements related to that category [40]. Descriptive statistical analysis of the child and parent characteristics was performed using IBM SPSS Statistics for Windows (version 28.0, Armonk, NY, USA: IBM Corp).

### Methodological rigor

To ensure methodological rigor, triangulation [45] was considered by using two methods of data collection and two researchers in the focus groups. Moreover, to avoid interpretation bias and increase credibility in the categorization process, multiple researchers were involved in the data analysis. The credibility of the research findings was strengthened by choosing illustrative quotes from a wide-spread of informants [46]. Reliability (dependability) [43] was achieved by making careful documentation of the categorization process in Microsoft Excel, enabling researchers to trace the source of the meaning unit in the transcripts of focus groups. Furthermore, all Microsoft Excel versions with changes throughout the data analysis were saved. To ensure reliability, a coding list (including explanations of the codes) was used to minimize cognitive change during the process of analysis. Generalization (transferability) [43, 47] was increased by stratifying the study sample by important disease-specific characteristics of EA and child age, in addition to providing a careful description of the setting and sample.

Finally, data saturation was evaluated *within* focus-groups, meaning that the field assistant during the focus group confirmed that all topics in the interview manual were covered in all five focus groups, and that all participants had the opportunity to contribute to the discussion. Data saturation was also determined *across* focus groups, by investigating if aerodigestive symptom experiences were discussed in all focus groups to confirm theoretical saturation [44].

## Results

### Study sample

Out of 30 parents who accepted study participation, 22 parents of children born with EA–TEF contributed as planned in five focus groups during the year 2024, in both non-clinical facilities at BCH (n = 4 focus groups, primary anastomosis/complex repair) and via Zoom video conference, specifically for ER participants (n = 1 focus group). The study sample is presented in Table 1. The open discussion lasted in total 7 h and 22 min (median 1,5 h, range 1 h and 1 min to 1 h and 51 min). Most parents were mothers (82%), had university/college education (94%) and represented children with different anatomical subtypes of EA–TEF. Although most children had peroral feeds (91%) at the time of the study, a subsample (28%) was also still tube fed.

**Table 1:** Presentation of characteristics of children born with esophageal atresia–tracheoesophageal fistula (n = 22) and one of their parents represented in the focus group study

Neonatal characteristics	n, %	Median (range)
Females	6 (27)	
Gestational weeks at birth		36 (28–40)
Birth weight (grams)^a^		2275 (1011–3634)
*Anatomical subtype*		
Isolated/pure EA (Gross A)	5 (23)	
EA with a proximal TEF (Gross B)	2 (9)	
EA with distal TEF (Gross C)	14 (64)	
TEF only (Gross E)	1 (4)	
*Associated anomalies*		
Yes, associated anomaly	16 (73)	
Cardiovascular	11 (50)	
Anorectal	3 (14)	
Uro-genital	4 (18)	
Limb	4 (18)	
-Vertebral	7 (32)	
*Surgical treatment(s)*		
Primary anastomosis/TEF repair	10 (45)	
Delayed primary anastomosis	3 (14)	
Foker procedure	4 (18)	
Esophageal replacement	5 (23)	
Posterior tracheopexy	9 (41)	
Major revisional surgery of esophagus, airways or both^b^	10 (45)	
Anti-reflux surgery	4 (18)	
*Feeding at follow-up*		
A gastrostomy		
Yes, previously	8(36)	
Yes, currently	5 (23)	
No	9 (41)	
Jejunostomy tube (J-tube)		
Yes, currently	1 (5)	
No	0	
Gastro-Jejunal (GJ) Tube		
Yes, previously	2 (9)	
Per oral feeding at follow-up	20 (91)	
*Parent characteristics*		
Mothers	18 (82)	
Age		39 (33–48)
Cohabitant partner	21 (95)	
University/college education	17 (94)^c^	
Born outside USA	2 (9)	

^a^Six missing values

^b^Includes five children who had esophageal replacements as revisional surgery, three after primary anastomosis, one after delayed primary anastomosis and one child after a Foker procedure, where eight children were referrals with need for revisional surgery after index operation outside study center

^c^Four missing values

### Symptom experiences

**Fig. 2:**
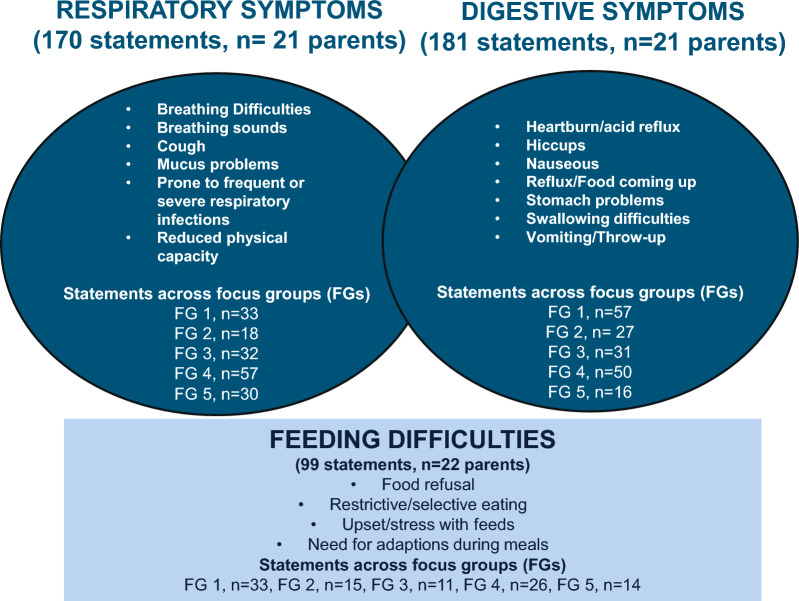
Illustration of a model showing categories of symptom experiences in children born with esophageal atresia–tracheoesophageal fistula aged 0,5–7 years as reported in five focus groups with their parents

Twenty-two parents made 450 unique statements about their children’s aerodigestive symptoms experiences. Figure 2 presents an overall model of their distribution into respiratory symptoms (n = 170 statements, n = 21 parents), digestive symptoms (181 statements, n = 21 parents) and feeding difficulties (99 statements, n = 22 parents) with its respective categories. Data saturation was confirmed as similar symptom experiences were discussed across all five focus groups (Fig. 2). Additionally, the field notes showed that all topics in the interview manual were covered and all participants could contribute to the discussion. Twenty parents (91%) described that their children had experienced difficulties that spanned across the main areas; respiratory symptoms, digestive symptoms and feeding difficulties.

### Respiratory symptom burden

#### Categories of respiratory symptom experiences

Table 2 lists the categories of their children’s respiratory symptoms in alphabetic order with its subcategories, demonstrating symptom expressions of Breathing difficulties (n = 21 subcategories), Breathing sounds (n = 6 subcategories), Cough (n = 17 subcategories), Mucus problems (n = 22 subcategories), Prone to frequent or severe respiratory infections (n = 20 subcategories) and Reduced physical capacity/strength (n = 8 subcategories) in young children with EA–TEF. As shown, parents’ statements indicated the most frequent child respiratory symptom experience was Cough (52 statements, 16 parents) and least frequent was Breathing sounds (11 statements, 4 parents).

**Table 2:** Presentation of categories and subcategories of respiratory symptom experiences in young children born with esophageal atresia–tracheoesophageal fistula in alphabetic order

Category-what symptom?	Breathing difficulties	Breathing sounds	Cough	Mucus problem	Prone to frequent or severe respiratory infections	Reduced physical capacity/strength
	(35 statements, 11 parents)	(10 statements, 4 parents)	(52 statements, 16 parents)	(34 statements, 16 parents)	(29 statements, 13 parents)	(10 statements, 7 parents)
Subcategory-symptom expression?	Body was working to breathe Chest retractions Desaturated Gasping for air Getting winded Go down completely (desaturated) Issues breathing Hard time breathing/Breathing difficulties Hyperventilating Need to get bagged Nighttime breathing difficulties Not breathing/not able to breath Out of breath Rapid breathing Respiratory distress Short of breath Slow down to gain his breath back Stopped breathing Turned blue Was blocked and muted Was muffled	Cracky sound in chest Raspy Junky Sounded like a bulldog when breathing Sounds like brewing when breathing Stridor	Barky cough Constant cough Cough (baseline) Cannot cough up stuff Cough that sounds loud Cough that sounds alarming Cough that sounds bad Cough sounding like croup Cough attack Creepy cough Death cough Distinct cough Heavy cough Junky Cough Night-time cough Persistent cough Raspy cough	Battle with mucus Build up Secretions Can't clear mucus Can't cough mucus up Congestion Difficulties cough up mucus Gag on mucus Getting mucus stuck Heavy mucus that comes up Mucus clearance issues Mucus problems Mucus lingers More mucus/increased secretions/more visible secretions Need to clear oneself out Need to cough up mucus Phlegm Produced a lot of mucus Secretions Spits out wads of mucus Takes a while/some days to get all mucus out Throw-up phlegm Worsening of mucus	Cannot clear infections good Difficulties getting better from sickness Frequent/Common respiratory infections Get respiratory infection easy Getting sick longer Gets sick fast Getting severely sick with colds Hot mess when he gets a cold ICU-needs due to respiratory infections Is laid low due to respiratory infections Hospital needs due to respiratory infections Longer respiratory infections Longer to recover More often sick More respiratory infections More severe respiratory infections Worsening in respiratory symptoms with colds Takes so much longer to get better The simplest of colds last forever Worsening at nights with respiratory infections	Does not have lot of strength/ Gets tired quick Is not a bruiser Les strength Less activity level Reduced physical capacity Slow Tiredness

#### Symptom frequency and severity experiences

Table 3 summarizes the six categories of respiratory symptom experiences with parents’ description of the symptom frequency and severity. Each respiratory symptom category was associated with specific situational contexts. Parents reported respiratory symptoms occurring in multiple context; during feeding (Breathing difficulties, Breathing sounds, Cough, Mucus problems, Reduced physical capacity/strength), during a respiratory infection (Breathing difficulties, Breathing sounds, Cough, Mucus problems), as part of children’s baseline status (Breathing sounds, Cough, Mucus problems), at exertion/exercise (Breathing difficulties, Cough, Reduced physical capacity/strength), and at night, during sleep or when laying down (Breathing difficulties, Cough). “My child was nursing, my child would get like a burst and their esophagus would fill up and it would crush their trachea. So, every time my child was feeding, they stopped breathing” (Informant 16, child age 2 years, complex repair)*.*

**Table 3:** An overview of the six categories of respiratory symptom experiences with parents’ description of the symptom frequency and severity in young children born with esophageal atresia–tracheoesophageal fistula

Symptom category with parent expressions of its frequency	When-situational context?	Symptom severity?			
		Amount/degree	Intensity	Care needs/consequences	Symptom progression
*Breathing difficulties* Always/constantly/every time/during feeding More often than other kids	Caring	A little bit/ A lot Bad/Worst/Severe Worse than compared to peers	Not reported	Need breaks Hospital care 911	Not reported
	Exertion/exercise				
	Feeding				
	Increased abdominal pressure (e.g. crying, laughing, pooping)				
	Nights/sleeping/laying down				
	Respiratory infection				
*Breathing sounds* Always during feeding All the time/always Every week Sometimes	Baseline/whenever	Really/much	Intense	Not reported	Not reported
	Feeding				
	Respiratory infection				
*Cough* Always/constant every time/constantly/always when was feeding All nights all the time/non-stop every night/every night Frequently/often/80% of the time Sometimes A few/at some point Remotely	Baseline/Whenever	Alarming A lot Really/Very bad	Attacks “Death” Heavy Persistent Takes so long to get better/will not go away	Looks from others Need suction School absence	Puke/Vomiting
	Exertion/exercise				
	Feeding				
	Nights/sleeping/laying down				
	Respiratory infection				
*Mucus problems* Always After every meal Frequently	Baseline/whenever	A lot/more/increased Heavy Spit out wads of	Persistent Present no matter what Will not go away	Change of diet Increased medical treatment Need suction	Cough Gag Puke/vomiting Respiratory infections
	Feeding				
	Respiratory infection				
	Strictures				
*Prone to frequent or severe respiratory infections* Not reported	Baseline/whenever	Sick fast/go downhill fast A lot of Bad With every cold	Persistent Longer time to recover	Emergency Room Increased medical treatments Hospital care Keep home—Avoid public places 911 School absence	Worse cough
	Seasonal/Winter				
	Starting daycare				
*Reduced energy/condition* Not reported	Exertion/exercise	Lower/worse compared to peers Lower/worse than sibling Lower/worse than younger kids Too tired	Not reported	Not reported	Not reported
	Feeding				

Considering the children’s symptom severity, parents also expressed multiple care needs/consequences of respiratory symptom experiences in their children. According to parents’ descriptions, Breathing difficulties and Being Prone to frequent or severe respiratory infections respectively, were symptom experiences leading to the child’s need for emergency and hospital care. “Anytime my child was sick they would go downhill really fast. And we only had to go in the hospital once for that” (Informant 11, child age 6 years, primary repair). Additionally, being Prone to frequent or severe respiratory symptoms was described to lead to additional consequences, including school absence. “My child missed so much school this year, we had pneumonia…The flu turned into pneumonia. My child had more than 19 absences.” (Informant 9, child age 5 years, primary repair)*.* Parents also described how their child’s Mucus problems could generate symptom progression of cough, gag, puke/vomiting and/or respiratory infections. “The mucus lingers and my child gets a lot of coughing of mucus and then that gags my child and then the vomiting happens” (Informant 22, child age 3 years, primary repair).

#### Symptom distress

**Fig. 3:**
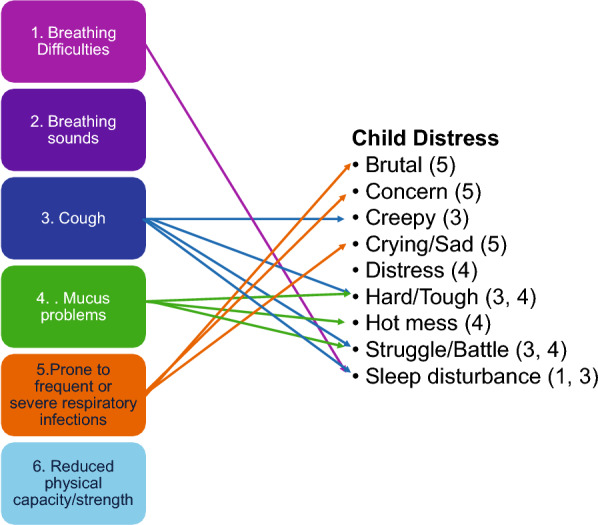
Illustration of categories of respiratory symptom experiences in children born with esophageal atresia–tracheoesophageal fistula aged 0,5–7 years and conceptual relationship to child distress as reported in focus groups with their parents

Figure 3 illustrates the conceptual link between the categories of respiratory symptom experiences and child distress. Four respiratory symptom categories (Breathing difficulties, Cough, Mucus problems, Prone to frequent or severe respiratory infections) were associated with child distress. “Just a few brutal months for them, where it just takes so much longer to get better and is a little more severe” (Informant 14, child age 2 years, primary repair)*.* Moreover, symptom distress included the children’s reactions of cry, showing distress and sleep disturbances. “The coughing, my child had a few little bouts where the coughing will keep my child up for a lot of the night. And that's hard.” (Informant 4, child age 1 year, primary repair)*.*

### Digestive symptom burden

#### Categories of digestive experiences

Table 4 lists the categories of their child’s digestive symptoms in alphabetic order with its subcategories, which encompassed symptom expressions of Acid reflux/heartburn (n = 7 subcategories), Hiccups (n = 1 subcategory), Nausea (n = 2 subcategories), Reflux/food coming up (n = 10 subcategories), Stomach problems (n = 4 subcategories), Swallowing difficulties (n = 24 subcategories) and Vomiting/throw-up (n = 6 subcategories) in young children with EA–TEF. As shown, the parents’ statements of the child’s digestive symptoms most frequently regarded the child’s Swallowing difficulties (97 statements, 20 parents) and least frequently Nausea (2 statements, 2 parents).

**Table 4:** The categories of their child’s digestive symptoms in alphabetic order with its subcategories

Category-what symptom?	Acid reflux/heartburn	Hiccups	Nausea	Reflux/food coming up	Stomach problems	Swallowing difficulties	Vomiting/throw-up
	(7 statements, 4 parents)	(3 statements, 3 parents)	(2 statements, 2 parents)	(24 statements, 13 parents)	(7 statements, 5 parents)	(97 statements, 20 parents)	(41 statements, 13 parents)
Subcategory-symptom expression?	A volcano in throat Burning feeling Complaints that it hurts Dragon in throat Neck that hurts Sour taste Throat that hurts	Having hiccups	Feel sick Feel nauseous	Cries when laying down at night and having reflux Food coming up/Something is coming up Difficulty keeping food down Having reflux Lots of reflux Making sounds when having reflux Nighttime reflux Regurgitate Struggle with reflux Wakes up at night due to reflux	Burpy Belly upset Haunted stomach Stomach ache /Belly ache	Can't clear crumbs Choking Cough with liquids/drinking Cough with every meal Cough on foods Cough on solids Difficulty drinking liquids Difficulty getting food up Difficulty getting food down Food not moving up or down Food getting stuck Food getting lodged Food not moving down Food never goes down Gags on foods/solids Gurgling noises Lengthy meals Liquids comes out of her trach Need to chew properly Problems swallowing solid foods Re-chewing foods Stuckies Spit up/Sputtering with feeds (chewing) Squirting sound move liquid down Vomiting/Throws-up with stuckies	Nighttime vomit Puke Spit up/Spit up large volume (after eating) Throw-up Vomit Worsening of vomiting

#### Symptom frequency and severity experiences

Table 5 gives an overview of the six categories of digestive symptom experiences with parents’ description of the symptom frequency and severity. A range of different symptom frequencies were described for Acid reflux/heartburn, Reflux/food coming up, Swallowing difficulties and Vomiting/throw-up, varying from always to seemingly random. “Sometimes my child makes a squirting sound trying to move liquids down” (Informant 1, child age 2 years, delayed TEF repair). All digestive symptoms as defined by its categories were described to occur with the child’s feeding. Additionally, the digestive symptoms were commonly reported to occur during Nights/sleep/laying down (Acid reflux/heartburn, Reflux/food coming up, Swallowing difficulties, Vomiting/throw-up) and during a Respiratory infection (Nausea, Swallowing difficulties, Vomiting/throw-up). “We're having, probably nighttime reflux events, with notable, notable vomit involved” (Informant 18, child age 5 years, esophageal replacement)*.*

**Table 5:** An overview of the categories of digestive symptom experiences with parents’ description of the symptom frequency and severity in young children born with esophageal atresia–tracheoesophageal fistula

Symptom category with parent expressions of its frequency	When-situational context?	Symptom severity?			
		Amount/degree	Intensity	Care needs/consequences	Symptom progression
*Acid reflux/Heartburn* Every once in a while	Feeding	Not reported	Not reported	Not reported	Not reported
	Nigths/sleeping/laying down				
*Hiccups* Not reported	Feeding	A lot of	Not reported	Not reported	Not reported
	Increased abdominal pressure (e.g. crying, laughing, pooping)				
*Nausea* Not reported	Feeding	Not reported	Not reported	Not reported	Not reported
	Respiratory infections				
*Reflux/food coming up* Constant/ongoing to this day Pretty rare Twice in four months	Feeding	A little bit Biggest difficulty Impactful Large volumes	Not reported	Not reported	Heartrate drops
	Nigths/sleeping/laying down				
*Stomach problems* Not reported	Feeding	A little bit	Not reported	Not reported	Not reported
*Swallowing difficulties* Countless times/constantly/all the time Sometimes Twice a week With every meal When eating normal age-appropriate diet	Feeding	A little bit Lots of problem Worst thing Difficult problem Manageable	Not reported	Don’t want to eat Hospital care 911 Need to chew properly Need to drink water anymore Adapt food intake	Becomes fussy Cough Drop sats Gagging Gurgling noise Hiccups Spit out Throw-up
	Nigths/sleeping/laying				
	Respiratory infections				
*Vomiting/throw-up* All the time/always Anything he ate At some point Every single day multiple times a day Every once in a while Every other night Very frequently Once or twice a day More often than not Multiple times Recurrently Two-to three times a day Two out three days a week Seemingly random, not super frequent	Baseline/whenever	Completely throws up/everything in his stomach Notable vomit Throws up a lot Throws up a little bit More than other children	Aggressive Projectile	Not reported	Not reported
	Feeding				
	Increased abdominal pressure (e.g. crying, laughing, pooping)				
	Nigths/sleeping/laying				
	Respiratory infection				

Considering the children’s experiences of symptom severity, Vomiting/throw-up was the only category characterized in terms of both amount/degree and intensity, whereas parents most commonly described their children’s Swallowing difficulties to lead to care needs/consequences and symptom progression. “Dealing with figuring out what foods are causing stuckies [when food gets lodged in the esophagus].”It's pretty frequent, the only way my child gets it out is by throwing up” (Informant 9, child age 5 years, primary repair)*.*

#### Symptom distress

**Fig. 4:**
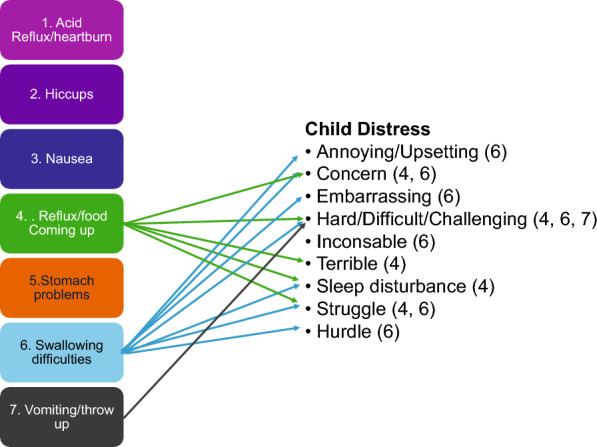
Illustration of categories of digestive symptom experiences in children born with esophageal atresia–tracheoesophageal fistula aged 0,5–7 years and conceptual relationship to child distress as reported in focus groups with their parents

Figure 4 illustrates the conceptual link between the categories of digestive symptom experiences and child distress. Three digestive symptom categories were associated with child distress (Reflux/food coming up, Swallowing difficulties, Vomiting/throw up). “When having a stuckie, my child gets super fussy, inconsolable”. (Informant 2, child age 1 year, complex repair)*.*

### Feeding difficulties

Table 6 lists the categories of their child’s feeding difficulties in alphabetic order with its subcategories; Food refusal (8 subcategories), Need for mealtime adjustment (7 subcategories), Selective/Restrictive eating (14 subcategories) and Upset/Stress with feeds (10 subcategories). For this category, parents most frequently described experiences of selective/restrictive eating, whereas Upset/Stress with feeds was least frequently described “Just having something in their mouth, really stresses my child out” (Informant 17, child age 7 years, esophageal replacement).

**Table 6:** The categories of their child’s feeding difficulties with its subcategories in alphabetic order

Category-what feeding difficulty?	Food refusal	Need for meal adaptations	Selective/restrictive eating	Upset/stress with feeds
	(18 statements, 9 parents)	(39 statements, 11 parents)	(31 statements, 15 parents)	(11 statements, 6 parents)
Subcategory-feeding difficulty expression?	Battle to take the bottle Completely quit eating Did not/never ate or refused eating Does not drink from a bottle No interest in eating Refused food Turning away from the bottle Wouldn't take much by mouth	Need for adjusted nipple size Need to drink or eat slowly Need for food cut up Need for increased fluid intake with swallowing Need for thickened liquid Need for tube feeding Need for adult watching the child when they are eating	Avoids food Couldn't eat Cannot eat all by mouth/do not eat by mouth Difficulties breastfeeding/bottle feeding Difficulty eating a certain amount Difficulty eating certain foods Doesn't feel hunger Does not eat regularly (through mouth) Eats only a few bites Eating was set back Hesitant towards small bites Not satisfied/hungry Picky/picky eater Pretend eating	Crying when nursing/crying when bottle-feeding Does not trust things in her mouth Fussy during breastfeeding Gets mad if being offered food Nervous eating Panic due to crumb or chunks in her mouth Stress when having something in her mouth Showed discomfort during feeding Spasm during feeds Upset during feeds

## Discussion

To the authors knowledge, this is the first focus group study with parents of children born with EA–TEF from the US, revealing that these children face a significant burden from respiratory and digestive symptom experiences as well as feeding difficulties during their first years of life. By using focus groups methodology, parent-reports uniquely shed light on their children’s symptom burden as a possible summative composite of the dimensions of frequency, severity and distress within these symptom areas.

### Respiratory symptom experiences

Although it is well known that children with EA–TEF can demonstrate restrictive and obstructive respiratory disease [48], this study responded to the lack of qualitative research regarding parents’ descriptions on their children’s respiratory symptoms experiences. Parents showed great ability to characterize the multiple ways respiratory symptoms took expression during their children’s first years of life. Accordingly, “cough” was a prominent aspect of EA–TEF in young children, which agrees with other studies with cough reported to occur in 95% of children with EA–TEF [49]. The high prevalence makes cough noteworthy, however, different studies have used varying definitions, such as the presence of cough [20, 49], chronic cough [21], cough > 4 weeks [50], continous cough[21], recurrent cough [21]. Defining the characteristics further may be important as different “coughs” may have different etiologies [13]. A typical professional categorization is dry and wet cough [13]. In two studies of children with EA–TEF, the authors evaluated cough during infections [21] and dry cough between colds [50]. However, parents in this study did not describe their children’s cough explicitly using “dry” or “wet” cough, which is consistent with findings of parents’ of children with cough in general [51]. Instead, their narratives prominently revealed different sounds of cough, day/night occurrence, intensity, and situations when cough occured, which could guide PROM development [19]. The significance of childhood cough has been recognized by the development of a chronic cough-specific QOL questionnaire, capturing its psychosocial implications [52]. However, this questionnaire was not adapted nor validated in children < 7 years, does not evaluate cough together with other respiratory symptoms or focus on sound or intensity characteristics. In our study, another major category of respiratory symptom experiences concerned the child’s experience of breathing difficulties, especially during feeding. In a recent study, apparent life-threatening events were observed in 22% of infants with EA–TEF (median 8 months of age) [53]. Such life-threatening airway collapse that leads to desaturation, bradycardia and sometimes loss of consciousness was also reported in our study and are typical during the child’s feeds. Together with mucus problems and being prone to frequent or severe respiratory infections, they suggest the presence of clinically significant TBM. In our study sample, 41% had a tracheopexy as a surgical management strategy for TBM, which highlight the high prevalence of severe TBM in our cohort and which can explain the significance of the child’s respiratory symptoms in this study population. However, across the five focus groups, seven out of the nine parents whose children received tracheopexy described an improvement of their child’s respiratory morbidity after tracheopexy. The parents’ perceived improvement of their child’s respiratory morbidity included a reduction of their child’s breathing difficulties, severe and/or frequent respiratory infections, cough, noisy breathing and/or mucus problems. Furthermore, our findings importantly highlight parents’ experiences of their child’s mucus problems as a root of symptom progression. In terms of symptom severity, breathing difficulties and being prone to frequent or severe respiratory infections were described as reasons of the child’s need for emergency care and hospitalization. Moreover, being prone to frequent or severe respiratory infections also led to a child’s school absence. In comparison, Swedish studies have shown that between 25% and 36% of children with EA–TEF are absent from school 12 times/year or more, and that this is related to respiratory morbidity and young child age [54, 55]. Additionally, parents in our study described choosing to keep their child home and avoid public places to prevent respiratory infections. This agrees with another study of parents of children with prophylactic antibiotics due to the risk of lower respiratory tract;. their parents described such social restrictions in the family [56]. In congruence with other studies, cough in children with EA–TEF led to experiences of stigmatization, uniquely the sound or character of the cough causing negative reactions from other people [33]. We also found that respiratory symptom experiences in children with EA–TEF were conceptually linked to child distress. In another study of children with LGEA aged 3–17 years [57], cough and airway infections were related to higher levels of mental health difficulties. Consequently, respiratory symptom experiences may have psychosocial consequences mirrored in the experience of symptom burden early in life of children with EA–TEF. Breathing sounds and reduced physical capacity/strength were least reported in this study, and may indicate less relevance to younger children. However, reduced physical capacity/strength may be valuable to understand further in depth, as a recent study showed that older children with EA–TEF have reduced physical activity levels than healthy controls [58]. The collective character of respiratory symptom burden in young children with EA–TEF highlight the need for preventative care strategies. Primary posterior tracheopexy at time of repair of EA has shown to reduce respiratory morbidity [59], but is recommended to be conducted at highly specialized centers [13]. A qualitative study from 2025 [60] included eight interviews with parents of children following treatment of TBM with aortopexy, where aortopexy was seen as life-saving and allowed a return to family life with better QOL.

### Digestive symptom experiences

In our study, the children’s symptom experiences of swallowing difficulties were frequently reported, consistent with other study findings [2, 10]. In this study, parents comprehensively described experiences of their child’s swallowing difficulties, reflecting multiple problems during the oro-pharyngeal and esophageal phases of swallowing [24]. Moreover, in the framework of symptom burden, symptoms of swallowing difficulties in young children with EA–TEF were described as a main reason for care needs/consequences, progression into new symptoms and child distress. Previously, swallowing difficulties have been shown to be associated with reduced HRQOL in children with EA–TEF aged 2–18 years [61, 62]. In comparison to previous studies, attention has also been brought to the negative relationship between swallowing difficulties in children with EA–TEF and parents’ or family impact respectively[63, 64]. Our findings can be explained by the context that parents of young children encounter nutritional intake issues on a daily basis [10], and may be negatively affected themselves by their child’s swallowing difficulties, which in turn could stimulate the discussion in focus groups. Despite its relevance to this population, several swallowing difficulty questionnaires used in children with EA–TEF, as described by Stewart et al. [24], have been developed for adults or for other conditions. The brief assessment tool EAT-10 has shown value in children with EA–TEF [65]. However, it was initially developed for adults, and items were not generated from patients/caregivers [66], as recommended for PROM development [19].

Additionally, rich descriptions of the child’s reflux/food coming up and vomiting/throw-up were provided by the parents, which can have different underlying reasons, GERD being one of them [31]. Interestingly, however, acid reflux/heartburn was reported by parents with the least frequency in our study and only reported for a child age 6 years or more. A possible explanation is that heartburn is difficult to observe for parents at first, and may be easier to understand when children can verbalize their sensations. In 2008, Malaty et al. [67] developed a psychometrically sound questionnaire for GERD in children aged 4–18 years, showing four main dimensions, namely symptoms, pain intensity, disability and satisfaction with health, but the items seemed not to be primarily generated from children or caregivers. Kleinmann et al. [68], demonstrated good reliability, validity and clinically responsive measure in GERD symptoms in infants. According to these findings, a measurement of GERD needs adaptation to different child ages.

In this study, symptom experiences like stomach-problems, hiccups and nausea were also described but to fewer extent. However, considering its possible existence, together with the other gastro-intestinal symptoms, the use of PedsQL Gastrointestinal Module [69, 70] which covers such symptom areas could help to establish a comprehensive understanding of frequency of digestive symptoms in young children with EA–TEF.

### Feeding difficulties

Feeding difficulties emerged as a distinct symptom category in young children with EA–TEF potentially indicating esophageal and respiratory dysfunction [2, 13]. As shown, young children with EA–TEF need multiple adaptations during meals. Despite their meal adaptations, our study findings revealed experiences of the children’s food refusal, restrictive/selective eating and being upset/stressed with feeds in early childhood years. Previous studies of children with EA–TEF have shown that they developed coping strategies to deal with nutritional intake situations from 2 years of age. With increased child age, they needed less social support and reached acceptance of their feeding difficulties. However, a subgroup of the children applied disengagement coping strategies, such as avoidance, emotional expression of fear/worry and distancing themselves from food/meals by e.g. hiding or throwing food away, and these children had worse HRQOL [71, 72]. This underlines the need for a psychometrically sound PROM of feeding difficulties that can be applied at an early childhood age after being born with EA–TEF. Feeding difficulties in children with EA–TEF have commonly been evaluated by using the Montreal Children’s Hospital Feeding Scale [25–27], which suits children aged 6 months to 6 years [73]. In comparison to content domains of feeding difficulties found in this study (Food refusal, Need for mealtime adjustement, Selective/Restrictive eating, Upset/Stress with feeds), it covers oral motor, oral sensory, appetite, maternal concern about feeding, mealtime behaviours, maternal strategies used and family reactions to their child’s feeding, suggesting that feeding difficulties as a psychometric construct can be differently defined. Recently, the parent-reported Eating Assessment Tool (Pedi-EAT), applicable from child age 6 months to 7 years was rigorously developed and content validated of a parent-report with input from researchers, clinicians, and parents [74]. Pedi-EAT measures Physiologic Symptoms, Problematic Mealtime Behaviors, Selective/Restrictive Eating, Oral Processing [74, 75], but use of Pedi-EAT in children with EA–TEF seems not to have been reported yet.

### Study strengths and limitations

This focus group study from the US is strengthened by responding to lack of research that gives voice to the needs of children born with EA–TEF. Our study proved data saturation [44] and contained more participants than other qualitative interview studies pertaining to EA–TEF [35–37]. However, focus groups findings cannot necessarily be generalized [42, 44]. The transferability of study findings may be impacted by BCH being a highly specialized center for esophageal and airway surgery, as such care may not be accessible for all children with EA–TEF. Additionally, BCH provides revisional surgery after failed attempts of surgical repair of EA–TEF at other institutions as well as it’s own. Another possible weakness is that our findings may be dependent on the moderator [42]. However, the approach is strengthened by a moderator who had no clinical relationship with the study participants and who used a semi-structured interview manual to standardize data collection. Furthermore, the level of intepretation of symptom experiences was manifest [43] to comply with current standards of PROM [19]. Still, manifest analysis does not unearth implicit meaning of symptom experiences, which is possible by alternative qualitative methods [43, 46]. From a qualitative perspective, quantification of participants and statements within categories is debatable [43], as each experience can be regarded as equally important [76]. However, quantification of categories has also been used to enrich the understanding of study findings [44]. Additionally, deductive analysis of symptom experiences led to symptom severity being subcategorized into four types of perceived severity. The definition of symptom severity has not been strict in previous literature [38–40],  but our findings correspond well with a child-generated definition of symptom severity as to “how bad” a symptom has been for them [77]. Our study is also strengthened by considerations of methodological rigor taken prior to the study [45, 46].

## Conclusions

During the first years of their lives, children born with EA–TEF experience a significant symptom burden as a possible summative composite of the dimensions respiratory and digestive symptom frequency, severity and distress, in addition to feeding difficulties. The reported symptom experiences strongly support the need for a disease-specific measurement of symptom burden in children with EA–TEF at an early child age, to be guided by the content and wording of parents’ descriptions and so help establish content validity for a disease-specific PROM of symptom burden. Future research should explore the equivalence with symptom experiences reported in older children born with EA–TEF, and iteratively test the patient-parent-generated items in psychometric evaluations. When finalized, such PROMs should be used in treatment evaluations to capture the patient's or parent’s perspectives on the effectiveness and impact of a treatment, complementing clinical assessment.

## Supplementary Information

Additional file 1.

Additional file 2.

## Data Availability

The datasets analyzed during the current study are available in the manuscript or in its additional files. Further information is not available to the public due to lack of ethical approval.

## Abbreviations

EA: Esophageal atresia
ER: Esophageal replacement
GERD: Gastro-esophageal reflux disease
HRQOL: Health-related Quality of Life
LGEA: Long-gap esophageal atresia
PRO: Patient-reported outcome
PROM: Patient-reported outcome measure
TEF: Tracheo-esophageal fistula
TBM: Tracheobronchomalacia
