# Supplementary material for: Experiences of symptom burden among young children born with esophageal atresia–tracheoesophageal fistula: a US focus group study
Source: Orphanet J Rare Dis. 2025 Aug 18;20:438. doi: 10.1186/s13023-025-03939-2 (PMC12362997; doi:10.1186/s13023-025-03939-2)
Supplement: Supplementary file 1 — Additional file 1. [file 13023_2025_3939_MOESM1_ESM.docx]

| **Additional file 1. Example of steps taken in the manifest content analysis of symptoms experiences described in focus group transcripts with parents of children born with esophageal atresia aged 0-7 years** | | | | | | | | |
| --- | --- | --- | --- | --- | --- | --- | --- | --- |
| **Meaning unit** | **Condensed meaning unit** | **Code** | **Subcategory** | **Category** | **When/situational context** | **Frequency** | **Severity** | **Distress** |
| And she's -- but it was like constant before, so when she -- before they did the tracheopexy, it was constant all the time. […] because she just constantly keeps […] But it is a persistent cough that's constantly just there. And I'm just like, you're not sick, but it's just there. | But it is a persistent cough that's constantly just there. And I'm just like, you're not sick, but it's just there. | Persistent cough that is constantly always there | Cough that is persistent or always there | Cough | Whenever/baseline | Constant | Intensity: persistent | Not Reported |
